# Effect of BCG vaccination on proinflammatory responses in elderly individuals

**DOI:** 10.1126/sciadv.abg7181

**Published:** 2021-08-04

**Authors:** Nathella Pavan Kumar, Chandrasekaran Padmapriyadarsini, Anuradha Rajamanickam, Shrinivasa B. Marinaik, Arul Nancy, Srinivasan Padmanaban, Nabila Akbar, Manoj Murhekar, Subash Babu

**Affiliations:** 1 ICMR-National Institute for Research in Tuberculosis, Chennai, India.; 2 ICMR-National Institute for Research in Tuberculosis– International Center for Excellence in Research, Chennai, India.; 3 ICMR-National Institute of Epidemiology, Chennai, India.

## Abstract

We investigated the influence of Bacillus Calmette-Guérin (BCG) vaccination on the unstimulated plasma levels of a wide panel of cytokines, chemokines, acute-phase proteins (APPs), matrix metalloproteinases (MMPs), and growth factors in a group of healthy elderly individuals (age, 60 to 80 years) at baseline (before vaccination) and 1 month after vaccination as part of our clinical study to examine the effect of BCG on COVID-19. Our results demonstrated that BCG vaccination resulted in diminished plasma levels of types 1, 2, and 17 and other proinflammatory cytokines and type 1 interferons. BCG vaccination also resulted in decreased plasma levels of CC, CXC chemokines, APPs, MMPs, and growth factors. Plasma levels of the aforementioned parameters were significantly lower in vaccinated individuals when compared to unvaccinated control individuals. Thus, our study demonstrates the immunomodulatory properties of BCG vaccination and suggests its potential utility in nonspecific vaccination of COVID-19 by down-modulating pathogenic inflammatory responses.

## Teaser

BCG vaccination significantly diminished proinflammatory responses in elderly individuals in COVID-19 hot spots.

## INTRODUCTION

The coronavirus disease (COVID-19) pandemic is a major public health crisis, and there is an important need to foster preventive and therapeutic strategies. Severe acute respiratory syndrome coronavirus 2 (SARS-CoV-2) is the contributory agent for COVID-19, and like the other respiratory coronaviruses, SARS-CoV-2 is spread mainly via respiratory droplets (*1*). Bacillus Calmette-Guérin (BCG) is a live-attenuated vaccine strain of *Mycobacterium bovis* used against tuberculosis (TB) (*2*). Previously published studies have demonstrated that BCG vaccine also engenders effective and broad protection against other respiratory diseases. Initial administration of the BCG vaccine lowers child mortality not due to TB (*3*, *4*). These nonspecific effects of BCG vaccination are not partial only to children, as vaccination also results in the decline of occurrence of respiratory tract infections in adolescents (*5*) and elderly individuals (*6*, *7*).

BCG vaccination is considered to confer a nonspecific rise in immunity (*8*), and this vaccine is known to act via both innate and adaptive immune responses (*9*). Commonly, during viral infections, a timely and strong innate immune response permits more rapid and efficient viral clearance and could even inhibit symptomatic infection or weaken the severity of the infection (*10*). BCG is thus being evaluated in various clinical trials for protection against SARS-CoV-2 infection and COVID-19 disease (*7*, *11*). However, the major concern with using BCG vaccination in hot spots of COVID-19 is the possibility of the vaccination inducing highly proinflammatory responses and thus worsening infection or disease in otherwise asymptomatic or mild cases. This is especially true in light of the fact that cytokine storm and other proinflammatory responses including high complement-reactive protein (CRP) levels are associated with bad prognosis and worse outcomes in this disease (*12*–*15*). Hence, we aimed to examine the interface between inflammation and BCG vaccination by evaluating a wide-ranging set of circulating inflammatory biomarkers before and after BCG vaccination in elderly individuals residing in hot spots for SARS-CoV-2 infection.

## RESULTS

### BCG vaccination results in diminished plasma levels of pro- and anti-inflammatory cytokines

**Fig. 1:**
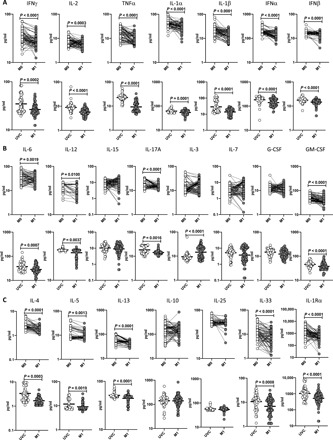
BCG vaccination results in diminished plasma levels of pro- and anti-inflammatory cytokines. (**A**) The plasma levels of type 1 cytokines, IL-1 family, and type 1 IFNs in BCG prevaccinated (M0) (*n* = 82) and M1 after vaccination (*n* = 82) and plasma levels of type 1 cytokines, IL-1 family, and type 1 IFNs in BCG-unvaccinated (UVC) (*n* = 55) and postvaccinated (M1) (*n* = 82) individuals are shown. (**B**) The plasma levels of proinflammatory cytokines in BCG prevaccinated (M0) (*n* = 82) and M1 after vaccination (*n* = 82) and plasma levels of proinflammatory cytokines in BCG-unvaccinated (UVC) (*n* = 55) and postvaccinated (M1) (*n* = 82) individuals are shown. (**C**) The plasma levels of anti-inflammatory cytokines in BCG prevaccinated (M0) (*n* = 82) and M1 after vaccination (*n* = 82) and plasma levels of anti-inflammatory cytokines in BCG-unvaccinated (UVC) (*n* = 55) and postvaccinated (M1) (*n* = 82) individuals are shown. The data are represented as scatter plots with each circle representing a single individual. For the analysis of M0 and M1, *P* values were calculated using the Wilcoxon matched-pair tests with Holm’s correction for multiple comparisons; for the analysis between UVC and M1, *P* values were calculated using the Mann-Whitney test with Holm’s correction for multiple comparisons.

Unstimulated plasma was used to examine the levels of types 1, 2, and 17, type 1 interferons (IFNs), and other proinflammatory cytokines following BCG vaccination; we compared the plasma levels of cytokines at baseline or before BCG vaccination [month 0 (M0)] and at month 1 (M1) after vaccination. As shown in Fig. 1A and table S1 (A to C), the type 1 cytokines IFNγ (*P* < 0.0001), interleukin-2 (IL-2) (*P* = 0.0003), and tumor necrosis factor–α (TNFα) (*P* < 0.0001), IL-1 family cytokines IL-1α (*P* < 0.0001) and IL-1β (*P* < 0.0001), and lastly, type 1 IFNs IFNα (*P* < 0.0001) and IFNβ (*P* = 0.0001); other proinflammatory cytokines IL-6 (*P* = 0.0004), IL-12 (*P* = 0.0010), IL-17A (*P* < 0.0001), and granulocyte-macrophage colony-stimulating factor (GM-CSF) (*P* < 0.0001) (Fig. 1B); and type 2 cytokines IL-4 (*P* < 0.0001), IL-5 (*P* = 0.0013), IL-13 (*P* < 0.0001), IL-33 (*P* < 0.0001), and IL-1Ra (*P* < 0.0001) (Fig. 1C), all showed significantly diminished levels at M1 compared to M0. Next, we compared the plasma levels of the aforementioned cytokines in postvaccinated individuals to unvaccinated controls. As shown in Fig. 1, BCG-vaccinated individuals exhibited decreased plasma levels of IFNγ, IL-2, TNFα, IL-1α, IL-1β, IFNα, IFNβ, IL-6, IL-12, IL-17A, GM-CSF, IL-4, IL-5, IL-13, IL-33, and IL-1Ra compared to unvaccinated individuals.

### BCG vaccination results in diminished plasma levels of CC and CXC chemokines

**Fig. 2:**
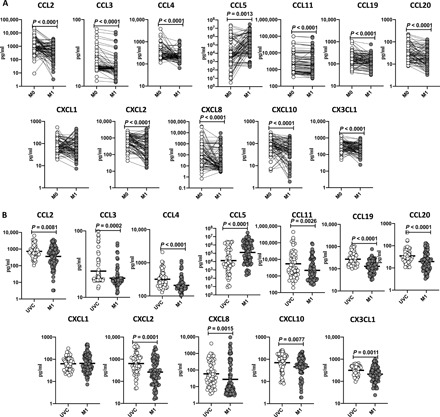
BCG vaccination results in diminished plasma levels of chemokines. (**A**) The plasma levels of CC chemokines in BCG prevaccinated (M0) (*n* = 64) and M1 after vaccination (*n* = 82) and plasma levels of CC chemokines in BCG-unvaccinated (UVC) (*n* = 55) and postvaccinated (M1) (*n* = 82) individuals are shown. (**B**) The plasma levels of CXC chemokines in BCG prevaccinated (M0) (*n* = 82) and M1 after vaccination (*n* = 82) and plasma levels of CXC chemokines in BCG-unvaccinated (UVC) (*n* = 55) and postvaccinated (M1) (*n* = 82) individuals are shown. The data are represented as scatter plots with each circle representing a single individual. For the analysis of M0 and M1, *P* values were calculated using the Wilcoxon matched-pair tests with Holms correction for multiple comparisons; for the analysis between UVC and M1, *P* values were calculated using the Mann-Whitney test with Holm’s correction for multiple comparisons.

Unstimulated plasma was used to determine the levels of CC and CXC chemokines following BCG vaccination; we compared the plasma levels of chemokines at baseline or before BCG vaccination (M0) and at M1 after vaccination. As shown in Fig. 2A and table S2 (A and B), the CC chemokines CCL2 (*P* < 0.0001), CCL3 (*P* < 0.0001), CCL4 (*P* < 0.0001), CCL5 (*P* = 0.0013), CCL11 (*P* < 0.0001), CCL19 (*P* < 0.0001), and CCL20 (*P* < 0.0001) and (Fig. 2B) CXC chemokines CXCL2 (*P* < 0.0001), CXCL8 (*P* < 0.0001), CXCL10 (*P* < 0.0001), and CX3CL1 (*P* < 0.0001) showed significantly diminished levels at M1 compared to M0. Next, we compared the plasma levels of CC and CXC chemokines in postvaccinated individuals to unvaccinated controls. As shown in Fig. 2, BCG-vaccinated individuals exhibited decreased plasma levels of CCL2, CCL3, CCL4, CCL5, CCL11, CCL19, 3, CCL20, CXCL1, CXCL2, CXCL8, CXCL10, and CX3CL1 compared to unvaccinated individuals.

### BCG vaccination results in diminished plasma levels of acute-phase proteins

**Fig. 3:**
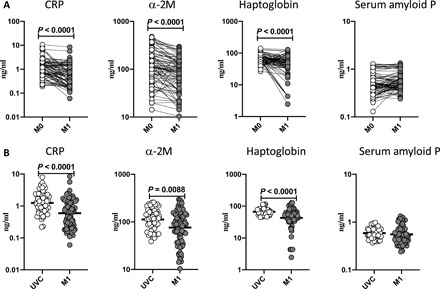
BCG vaccination results in diminished plasma levels of APPs. The plasma levels of APPs in BCG prevaccinated (M0) (*n* = 82) and M1 after vaccination (*n* = 82) are shown. Data are shown as line diagrams with each line representing a single individual. *P* values were calculated using the Wilcoxon matched-pair tests with Holm’s correction for multiple comparisons. The plasma levels of APPs in BCG-unvaccinated (UVC) (*n* = 55) and postvaccinated (M1) (*n* = 82) individuals are also shown. The data are represented as scatter plots with each circle representing a single individual. *P* values were calculated using the Mann-Whitney test with Holm’s correction for multiple comparisons.

We used unstimulated plasma to elucidate the levels of acute-phase proteins (APPs) following BCG vaccination and compared the plasma levels of APPs at baseline or before BCG vaccination (M0) and at M1 after vaccination. As shown in Fig. 3 and table S3, CRP (*P* < 0.0001), alpha-2 macroglobulin (a-2M) (*P* < 0.0001), and haptoglobin (*P* < 0.0001) showed significantly diminished levels at M1 compared to M0. Next, we compared the circulating levels of APPs in postvaccinated individuals to unvaccinated controls. As shown in Fig. 3, BCG-vaccinated individuals exhibited decreased frequencies of CRP, a-2M, and haptoglobin compared to unvaccinated individuals.

### BCG vaccination results in diminished plasma levels of matrix metalloproteinases and growth factors

**Fig. 4:**
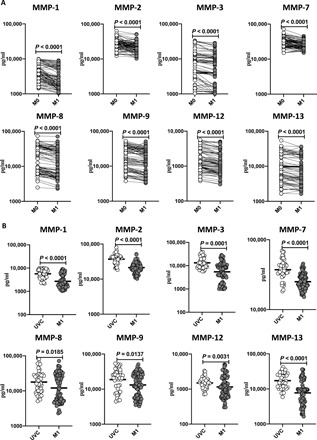
BCG vaccination results in diminished plasma levels of MMPs. The plasma levels of MMPs in BCG prevaccinated (M0) (*n* = 82) and M1 after vaccination (*n* = 82) are shown. Data are shown as line diagrams with each line representing a single individual. *P* values were calculated using the Wilcoxon matched-pair tests with Holm’s correction for multiple comparisons. The plasma levels of MMPs in BCG-unvaccinated (UVC) (*n* = 55) and postvaccinated (M1) (*n* = 82) individuals are also shown. The data are represented as scatter plots with each circle representing a single individual. *P* values were calculated using the Mann-Whitney test with Holm’s correction for multiple comparisons.

**Fig. 5:**
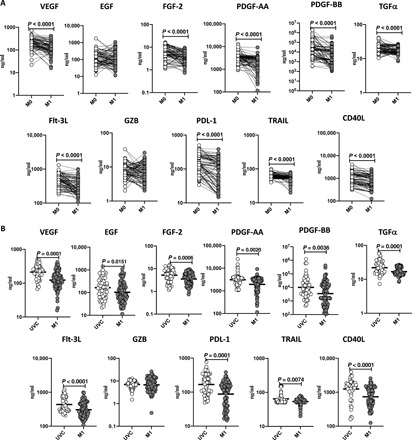
BCG vaccination results in diminished plasma levels of growth factors. The plasma levels of growth factors in BCG prevaccinated (M0) (*n* = 82) and M1 after vaccination (*n* = 82) are shown. Data are shown line diagrams with each line representing a single individual. *P* values were calculated using the Wilcoxon matched-pair tests with Holm’s correction for multiple comparisons. The plasma levels of MMPs in BCG-unvaccinated (UVC) (*n* = 55) and postvaccinated (M1) (*n* = 82) individuals are also shown. The data are represented as scatter plots with each circle representing a single individual. *P* values were calculated using the Mann-Whitney test with Holm’s correction for multiple comparisons.

To examine the unstimulated plasma levels of matrix metalloproteinases (MMPs) and growth factors following BCG vaccination, we compared the plasma levels of MMPs and growth factors at baseline or before BCG vaccination (M0) and at M1 after vaccination. As shown in Fig. 4 and table S4, MMP-1 (*P* < 0.0001), MMP-2 (*P* < 0.0001), MMP-3 (*P* < 0.0001), MMP-7 (*P* < 0.0001), MMP-8 (*P* < 0.0001), MMP-9 (*P* < 0.0001), MMP-12 (*P* < 0.0001), and MMP-13 (*P* < 0.0001) showed significantly diminished levels at M1 compared to M0. As shown in Fig. 5 and table S5, vascular endothelial growth factor (VEGF) (*P* < 0.0001), fibroblast growth factor 2 (FGF-2) (*P* < 0.0001), platelet-derived growth factor–AA (PDGF-AA) (*P* < 0.0001) and PDGF-BB (*P* < 0.0001), transforming growth factor–α (TGF-α) (*P* < 0.0001), Flt-3L (*P* < 0.0001), programmed death ligand–1 (PDL-1) (*P* < 0.0001), tumor necrosis factor-related apoptosis-inducing ligand (TRAIL) (*P* < 0.0001), and CD40L (*P* < 0.0001) showed significantly diminished levels at M1 compared to M0. Next, we compared the plasma levels of MMPs and growth factors in postvaccinated individuals to unvaccinated controls. As shown in Fig. 4, BCG-vaccinated individuals exhibited decreased plasma levels of MMP-1, MMP-2, MMP-3, MMP-7, MMP-8, MMP-9, MMP-12, and MMP-13 compared to unvaccinated individuals. As shown in Fig. 5, BCG-vaccinated individuals exhibited decreased plasma levels of VEGF, FGF-2, PDGF-AA, PDGF-BB, TGF-α, Flt-3L, PDL-1, TRAIL, and CD40L compared to unvaccinated individuals.

## DISCUSSION

BCG is considered to be one of the most broadly used vaccines in the world, with around 130 million infants receiving this vaccine every year (*16*). There is improved interest in BCG vaccination of elderly population, especially in countries with high number of reported COVID-19 cases. Immunity produced by some already available vaccines such as BCG has been recommended to be used as a conceivable protective approach against COVID-19 to bridge the period until a specific vaccine is available (*16*). Previous studies have reported that BCG vaccination may confer protection against respiratory tract infections, involving viral infections, and hence, in general, BCG vaccination might be an effective prophylactic measure against SARS-CoV-2 infection and/or might reduce disease severity (*16*, *17*). The current finding determined that BCG vaccination led to a lowering of the systemic levels of inflammatory markers after vaccination claiming for a protective effect.

Elderly individuals with comorbidities, such as hypertension, diabetes, or heart diseases, are at bigger risk of developing severe COVID-19 (*15*, *18*, *19*), demonstrating that a weakened innate antiviral immune response may lead to SARS-CoV-2 susceptibility. Recent published studies have stated that BCG vaccination–mediated trained immunity enhances antiviral immune responses and, in addition, that BCG-induced trained immunity could be a competent preventive measure against SARS-CoV-2 infection and COVID-19 severity (*16*, *20*, *21*). It also still remains unknown as to how BCG reduces overall inflammation while at the same time improving myeloid and heterologous T cell responsiveness.

It has been clearly reported that cytokines play a key role in immunopathology during viral infection. Recent published studies have reported that elevated inflammatory cytokine (such as TNFα, IL-1β, IL-6, IL-10, IL-17, IL-18, and IFNγ) levels were seen in active COVID-19 cases compared to healthy donors (*22*). TNFα and IFNγ are known to particularly drive COVID-19 disease severity (*14*), and in addition, IL-6, IL-1β, and IL-12 have been consistently implicated in severe disease (*22*). In this study, we aimed to understand the function of inflammatory cytokines on BCG-vaccinated individuals before and 1 month after vaccination in COVID-19 hot spots. Our current findings report that induction of the BCG vaccine–induced inflammatory cytokine response is clearly dampened in elderly individuals at 1 month. This finding corroborates the recently published clinical results of ACTIVATE (a randomized clinical trial for enhanced trained immune responses through BCG vaccination to prevent infections of the elderly), which suggest that BCG vaccination is safe and reduces the number of infections in an elderly population at risk (*7*). In addition, our finding also corroborates another study, which clearly reveals that BCG vaccination down-regulates circulating inflammatory markers (*23*). Moreover, apart from a variety of proinflammatory cytokines (which could possibly play a detrimental role in COVID-19), anti-inflammatory responses including IL-10 and IL-33, which have been implicated in COVID-19 disease severity, were also decreased in vaccinated individuals (*24*, *25*).

The activation of the immune system plays a fundamental role in defending against infectious agents, and it is been complemented by inflammatory mediator release (*26*). Like cytokines, chemokines are also an important inflammatory mediator in regulating the disease during the viral infection (*27*). In this study, we determined the circulating levels of CC and CXC chemokines, and our results clearly revealed that both CC and CXC chemokines are significantly decreased after 1 month of BCG after vaccination, indicating that BCG dampens proinflammatory chemokine responses as well. Recently published studies have also described that chemokines such as CXCL10 and CCL7 are elevated SARS-CoV-2 infection and are associated with disease severity (*28*). Thus, in addition to cytokines, proinflammatory responses in the form of systemic chemokines are also dampened in BCG-vaccinated elderly individuals.

Systemic inflammation is typically characterized by elevations in the levels of APPs, including CRP, a-2M, haptoglobin, and serum amyloid P. Recent studies have reported that there is a higher concentration of inflammatory markers such as CRP in patients with severe or moderate SARS-CoV-2 infection and during the longitudinal follow-up levels of CRP steadily declined within 10 days after admission in moderate and severe cases, indicating that CRP can be used as the potent inflammatory biomarker (*29*). In this study, we wanted to determine the effect of BCG vaccination on APPs and report that CRP, a-2M, and haptoglobin were significantly diminished, indicating that, upon BCG vaccination, the inflammatory responses are significantly modulated.

MMPs are discharged during lung inflammation in the extracellular matrix that leads to elevated chemokines with inflammatory properties (*30*). Published studies clearly demonstrated that MMP-3 and MMP-9 were significantly elevated and also act as immune markers for inflammation in patients with COVID-19 (*31*, *32*). In this study, we demonstrate that almost all the estimated MMPs, MMP-1, MMP-2, MMP-3, MMP-7, MMP-8, MMP-9, MMP-12, and MMP-13, were significantly down-regulated at 1 month after vaccination when compared to baseline. Thus, BCG is likely to potentially limit any damaging inflammatory responses induced in the lung during COVID-19 as well. Growth factors and their receptors are known to be involved in the process of viral infection. Not many growth factors except VEGF appear to be an essential player in the pathogenesis of many viral diseases including SARS-CoV-2 (*33*, *34*). Few studies have reported that, among the VEGF family subtype, VEGF-D was recognized as the main indicator linked to the severity of COVID-19 (*35*). In our study, many of the measured growth factors were significantly diminished in 1-month BCG postvaccinated individuals compared to baseline, indicating that even growth factors associated with inflammation are dampened.

In conclusion, the current study emphasizes that the effect of BCG vaccination is safe and does not lead to increased inflammation in elderly individuals. The results from this study not only corroborate the immunomodulatory properties of BCG vaccination but also reveal a clear effect of (non)specific immunogenicity of BCG vaccination on systemic inflammation. These results suggest that BCG could potentially act to inhibit the biomarkers of inflammation by the immune cells in the blood, but this remains to be proven. In addition, it is still unknown as to how long this inhibitory effect can persist. In this study, we report that recent BCG vaccination was not associated with hyperinflammation but was, in turn, associated with down-modulated basal inflammatory status, which might play a protective role in elderly population against inflammatory diseases. In addition, it is quite possible that pathogen-specific immune responses are not affected by BCG vaccination or, in contrast, actually enhanced. We do not have a placebo control group in our study (only unvaccinated controls at baseline), and this is a major limitation. Our findings also provide a mechanistic explanation for the findings in which BCG protected against or improved the outcomes in inflammatory, allergic, or autoimmune diseases. An improved understanding of these results may support the vaccine efficacy and explore innovative applications of BCG vaccination.

## MATERIALS AND METHODS

### Ethics statement

The study was approved by the ethics committees of National Institute for Research in Tuberculosis (NIRT) (NIRT-INo:2020010). Informed written consent was obtained from all participants. The study is part of the clinical trial entitled “Study to evaluate the effectiveness of BCG vaccine in reducing morbidity and mortality in elderly individuals in COVID-19 hotspots in India.” The study was also registered in clinical trial registry (NCT04475302).

### Study population

To study the immunological effects of BCG vaccination, *n* = 82 elderly individuals, between 60 and 80 years, residing in hot spots for SARS-CoV-2 infection were included in the study between June 2020 and October 2020 in Chennai, India after obtaining informed consent from the study participants. Eighty-two participants received a single dose of BCG vaccine (freeze-dried) manufactured by Serum Institute of India, Pune. The adult dose of BCG vaccine was 0.1 ml, injected intradermally over the distal insertion of the deltoid muscle onto the left humerus (approximately one-third down the left upper arm). Elderly individuals (*n* = 55) not vaccinated from the same hot spot area were considered as controls. Demographic profile of study population is described in Table 1. Blood was drawn from the vaccinated participants at baseline (before vaccination) and at 1 month following vaccination. Main exclusion criteria were elderly population positive for SARS-CoV-2 infection by either antibody (serology) or polymerase chain reaction test; known HIV, malignancy, transplant recipient, or on dialysis; recently (in the last 6 months) diagnosed with TB or currently on anti-TB treatment or antipsychiatric medications; and any BCG vaccine contraindication like allergy or hypersensitivity to BCG.

**Table 1:** Demographic profile of the study population. SpO2%, oxygen saturation.

	**BCG-vaccinated**		**BCG-** **unvaccinated**
**Participants enrolled**	***n* = 82**		***n* = 55**
	**M0 (*n* = 82)**	**M1 (*n* = 82)**	
Age (median)	66 (60–78)		65 (60–80)
Gender (M/F)	50/32		34/21
Height (median)	158 cm		155 cm
Weight (median)	61.4 kg		63 kg
Pulse rate (median)	88		88
Systolic blood pressure (median)	140		140
Diastolic blood pressure (median)	82		80
SpO2% (median)	98		98
Smoking, no. (%)	3 (3.6%)		3 (2%)
Alcoholism, no. (%)	5 (6.1%)		3 (2%)
Diabetes mellitus	25 (30.5%)		29 (52%)
Cardiovascular disease, no. (%)	10 (12.2%)		9 (16%)
Respiratory diseases, no. (%)	8 (9.8%)		7 (12%)
Musculoskeletal disease, no. (%)	0		1 (1%)
Gastrointestinal, no. (%)	1 (1.2%)		0
Genitourinary, no. (%)	2 (2.4%)		1 (1%)
Endocrine, no. (%)	35 (42.7%)		17 (30%)
Hematological, no. (%)	0		0
Neoplasia, no. (%)	0		0
Dermatological, no. (%)	6 (7.3%)		1 (1%)
Neurological, no. (%)	0		1 (1%)
Psychological, no. (%)	0		0
Allergies, no. (%)	2 (2.4%)		0

### Multiplex assays

Circulating plasma levels of APPs, cytokines, chemokines, and MMPs were measured using the Luminex MAGPIX Multiplex Assay system (Bio-Rad, Hercules, CA). MILLIPLEX MAP Human Cardiovascular Disease (Acute Phase) Magnetic Bead Panel 3 was used to measure the APPs, Luminex Human Magnetic Assay Kit 45 Plex (R&D Systems) was used to measure the cytokines and chemokine levels, and Luminex Human Magnetic MMP Assay Kit 8 Plex (R&D Systems) was used to measure the MMP levels. The lowest detection limits for APPs was as follows: a-2M, 0.49 ng/ml; CRP, 0.05 ng/ml; haptoglobin, 0.06 ng/ml; and serum amyloid A-1, 0.06 ng/ml. The lowest detection limits for cytokines were as follows: IFNγ, 5.7 pg/ml; IL-2, 3.6 pg/ml; TNFα, 12.4 pg/ml; IL-1α, 10.6 pg/ml; IL-1β, 3.5 pg/ml; IFNα, 3.9 pg/ml; IFNβ 3.25 pg/ml; IL-6, 9.0 pg/ml; IL-12, 18.5 pg/ml; IL-15, 2.5 pg/ml; IL-17A, 9 pg/ml; IL-3, 17 pg/ml; IL-7, 3.5 pg/ml; G-CSF, 8.4 pg/ml; GM-CSF, 18.4 pg/ml; IL-4, 1.1 pg/ml; IL-5, 6.2 pg/ml; IL-13, 31.8 pg/ml; IL-10, 32.2 pg/ml; IL-25, 18.4 pg/ml; IL-33, 13.8 pg/ml; and IL-1Ra, 11.7 pg/ml. The lowest detection limits for chemokines were as follows: CCL2, 5.9 pg/ml; CCL3, 5.1 pg/ml; CCL4, 103.8 pg/ml; CCL5, 297 pg/ml; CCL11, 21.6 pg/ml; CCL19, 3.9 pg/ml; CCL20, 2.4 pg/ml; CXCL1, 19.1 pg/ml; CXCL2, 21.1 pg/ml; CXCL8, 1.4 pg/ml; CXCL10, 2.6 pg/ml; and CX3CL1, 188 pg/ml. The lowest detection limits for MMPs and growth factors were as follows: MMP-1, 23.87 pg/ml; MMP-2, 91.7 pg/ml; MMP-3, 77.9 pg/ml; MMP-7, 78.4 pg/ml; MMP-8, 84.9 pg/ml; MMP-9, 118.3 pg/ml; MMP-12, 9.2 pg/ml; and MMP-13, 211.3 pg/ml. VEGF, 5.9 pg/ml; EGF, 8.6 pg/ml; FGF-2, 8.7 pg/ml; PDGF-AA, 5.2 pg/ml; PDGF-BB, 7.31 pg/ml; TGFa, 8.6 pg/ml; Flt-3 L, 22.9 pg/ml; granzyme B, 4.9 pg/ml; PDL-1, 69.3 pg/ml; and TRAIL, 22.5 pg/ml.

### Statistical analysis

Geometric means were used for measurements of central tendency. Wilcoxon signed-rank test was used to compare the levels of inflammatory markers in BCG-vaccinated group at M0 and M1. Statistically significant differences between unvaccinated and BCG-vaccinated M1 groups were analyzed using the Mann-Whitney test. Analyses were performed using GraphPad Prism version 9.0.

## SUPPLEMENTARY MATERIALS

Supplementary material for this article is available at http://advances.sciencemag.org/cgi/content/full/7/32/eabg7181/DC1

## Appendix

View/request a protocol for this paper from *Bio-protocol*.

## References

[1] M. Z. Tay, C. M. Poh, L. Renia, P. A. MacAry, L. F. P. Ng, The trinity of COVID-19: Immunity, inflammation and intervention. Nat. Rev. Immunol. 20, 363–374 (2020). 32346093 10.1038/s41577-020-0311-8 PMC7187672

[2] C. S. Benn, M. G. Netea, L. K. Selin, P. Aaby, A small jab–a big effect: Nonspecific immunomodulation by vaccines. Trends Immunol. 34, 431–439 (2013). 23680130 10.1016/j.it.2013.04.004

[3] S. Biering-Sorensen, P. Aaby, N. Lund, I. Monteiro, K. J. Jensen, H. B. Eriksen, F. Schaltz-Buchholzer, A. S. P. Jorgensen, A. Rodrigues, A. B. Fisker, C. S. Benn, Early BCG-Denmark and neonatal mortality among infants weighing <2500 g: A randomized controlled trial. Clin. Infect. Dis. 65, 1183–1190 (2017). 29579158 10.1093/cid/cix525 PMC5849087

[4] M. L. Garly, C. L. Martins, C. Bale, M. A. Balde, K. L. Hedegaard, P. Gustafson, I. M. Lisse, H. C. Whittle, P. Aaby, BCG scar and positive tuberculin reaction associated with reduced child mortality in West Africa. A non-specific beneficial effect of BCG? Vaccine 21, 2782–2790 (2003). 12798618 10.1016/s0264-410x(03)00181-6

[5] E. Nemes, H. Geldenhuys, V. Rozot, K. T. Rutkowski, F. Ratangee, N. Bilek, S. Mabwe, L. Makhethe, M. Erasmus, A. Toefy, H. Mulenga, W. A. Hanekom, S. G. Self, L.-G. Bekker, R. Ryall, S. Gurunathan, C. A. DiazGranados, P. Andersen, I. Kromann, T. Evans, R. D. Ellis, B. Landry, D. A. Hokey, R. Hopkins, A. M. Ginsberg, T. J. Scriba, M. Hatherill; C-040-404 Study Team, Prevention of *M. tuberculosis* infection with H4:IC31 vaccine or BCG revaccination. N. Engl. J. Med. 379, 138–149 (2018). 29996082 10.1056/NEJMoa1714021 PMC5937161

[6] E. Wardhana, A. Datau, A. Sultana, V. V. V. Mandang, E. Jim, The efficacy of Bacillus Calmette-Guerin vaccinations for the prevention of acute upper respiratory tract infection in the elderly. Acta Med. Indones. 43, 185–190 (2011). 21979284

[7] E. J. Giamarellos-Bourboulis, M. Tsilika, S. Moorlag, N. Antonakos, A. Kotsaki, J. Dominguez-Andres, E. Kyriazopoulou, T. Gkavogianni, M. E. Adami, G. Damoraki, P. Koufargyris, A. Karageorgos, A. Bolanou, H. Koenen, R. van Crevel, D. I. Droggiti, G. Renieris, A. Papadopoulos, M. G. Netea, ACTIVATE: Randomized clinical trial of BCG vaccination against infection in the elderly. Cell 183, 315–323.e9 (2020). 32941801 10.1016/j.cell.2020.08.051 PMC7462457

[8] C. Covian, A. Fernández-Fierro, A. Retamal-Diaz, F. E. Díaz, A. E. Vasquez, M. K. Lay, C. A. Riedel, P. A. Gonzalez, S. M. Bueno, A. M. Kalergis, BCG-induced cross-protection and development of trained immunity: Implication for vaccine design. Front. Immunol. 10, 2806 (2019). 31849980 10.3389/fimmu.2019.02806 PMC6896902

[9] B. Freyne, A. Marchant, N. Curtis, BCG-associated heterologous immunity, a historical perspective: Intervention studies in animal models of infectious diseases. Trans. R. Soc. Trop. Med. Hyg. 109, 287 (2015). 25770252 10.1093/trstmh/trv021

[10] V. C. Lam, L. L. Lanier, NK cells in host responses to viral infections. Curr. Opin. Immunol. 44, 43–51 (2017). 27984782 10.1016/j.coi.2016.11.003 PMC5451301

[11] M. Noval Rivas, J. E. Ebinger, M. Wu, N. Sun, J. Braun, K. Sobhani, J. E. Van Eyk, S. Cheng, M. Arditi, BCG vaccination history associates with decreased SARS-CoV-2 seroprevalence across a diverse cohort of healthcare workers. J. Clin. Invest. 131, e145157 (2021). 10.1172/JCI145157 PMC7810479 33211672

[12] V. J. Costela-Ruiz, R. Illescas-Montes, J. M. Puerta-Puerta, C. Ruiz, L. Melguizo-Rodriguez, SARS-CoV-2 infection: The role of cytokines in COVID-19 disease. Cytokine Growth Factor Rev. 54, 62–75 (2020). 32513566 10.1016/j.cytogfr.2020.06.001 PMC7265853

[13] Q. Ye, B. Wang, J. Mao, The pathogenesis and treatment of the “Cytokine Storm” in COVID-19. J. Infect. 80, 607–613 (2020). 32283152 10.1016/j.jinf.2020.03.037 PMC7194613

[14] F. Zhang, J. R. Mears, L. Shakib, J. I. Beynor, S. Shanaj, I. Korsunsky, A. Nathan; Accelerating Medicines Partnership Rheumatoid Arthritis and Systemic Lupus Erythematosus (AMP RA/SLE) Consortium, L. T. Donlin, S. Raychaudhuri, IFN-γ and TNF-α drive a *CXCL10*+ *CCL2*+ macrophage phenotype expanded in severe COVID-19 lungs and inflammatory diseases with tissue inflammation. Genome Med. 13, 64 (2021). 33879239 10.1186/s13073-021-00881-3 PMC8057009

[15] W.-J. Guan, W.-H. Liang, Y. Zhao, H.-R. Liang, Z.-S. Chen, Y.-M. Li, X.-Q. Liu, R.-C. Chen, C.-L. Tang, T. Wang, C.-Q. Ou, L. Li, P.-Y. Chen, L. Sang, W. Wang, J.-F. Li, C.-C. Li, L.-M. Ou, B. Cheng, S. Xiong, Z.-Y. Ni, J. Xiang, Y. Hu, L. Liu, H. Shan, C.-L. Lei, Y.-X. Peng, L. Wei, Y. Liu, Y.-H. Hu, P. Peng, J.-M. Wang, J.-Y. Liu, Z. Chen, G. Li, Z.-J. Zheng, S.-Q. Qiu, J. Luo, C.-J. Ye, S.-Y. Zhu, L.-L. Cheng, F. Ye, S.-Y. Li, J.-P. Zheng, N.-F. Zhang, N.-S. Zhong, J.-X. He; China Medical Treatment Expert Group for COVID-19, Comorbidity and its impact on 1590 patients with COVID-19 in China: A nationwide analysis. Eur. Respir. J. 55, 2000547 (2020). 32217650 10.1183/13993003.00547-2020 PMC7098485

[16] M. G. Netea, E. J. Giamarellos-Bourboulis, J. Dominguez-Andres, N. Curtis, R. van Crevel, F. L. van de Veerdonk, M. Bonten, Trained immunity: A tool for reducing susceptibility to and the severity of SARS-CoV-2 infection. Cell 181, 969–977 (2020). 32437659 10.1016/j.cell.2020.04.042 PMC7196902

[17] S. J. C. F. M. Moorlag, R. J. W. Arts, R. van Crevel, M. G. Netea, Non-specific effects of BCG vaccine on viral infections. Clin. Microbiol. Infect. 25, 1473–1478 (2019). 31055165 10.1016/j.cmi.2019.04.020

[18] S. Richardson, J. S. Hirsch, M. Narasimhan, J. M. Crawford, T. McGinn, K. W. Davidson; Northwell COVID-19 Research Consortium, D. P. Barnaby, L. B. Becker, J. D. Chelico, S. L. Cohen, J. Cookingham, K. Coppa, M. A. Diefenbach, A. J. Dominello, J. Duer-Hefele, L. Falzon, J. Gitlin, N. Hajizadeh, T. G. Harvin, D. A. Hirschwerk, E. J. Kim, Z. M. Kozel, L. M. Marrast, J. N. Mogavero, G. A. Osorio, M. Qiu, T. P. Zanos, Presenting characteristics, comorbidities, and outcomes among 5700 patients hospitalized with COVID-19 in the New York City area. JAMA 323, 2052–2059 (2020). 32320003 10.1001/jama.2020.6775 PMC7177629

[19] E. K. Stokes, L. D. Zambrano, K. N. Anderson, E. P. Marder, K. M. Raz, S. El Burai Felix, Y. Tie, K. E. Fullerton, Coronavirus disease 2019 case surveillance—United States, January 22-May 30, 2020. MMWR Morb. Mortal. Wkly Rep. 69, 759–765 (2020). 32555134 10.15585/mmwr.mm6924e2 PMC7302472

[20] L. A. J. O’Neill, M. G. Netea, BCG-induced trained immunity: Can it offer protection against COVID-19? Nat. Rev. Immunol. 20, 335–337 (2020). 32393823 10.1038/s41577-020-0337-y PMC7212510

[21] T. O. Kleen, A. A. Galdon, A. S. MacDonald, A. G. Dalgleish, Mitigating coronavirus induced dysfunctional immunity for at-risk populations in COVID-19: Trained immunity, BCG and “New Old Friends”. Front. Immunol. 11, 2059 (2020). 33013871 10.3389/fimmu.2020.02059 PMC7498663

[22] D. M. Del Valle, S. Kim-Schulze, H. H. Huang, N. D. Beckmann, S. Nirenberg, B. Wang, Y. Lavin, T. H. Swartz, D. Madduri, A. Stock, T. U. Marron, H. Xie, M. Patel, K. Tuballes, O. Van Oekelen, A. Rahman, P. Kovatch, J. A. Aberg, E. Schadt, S. Jagannath, M. Mazumdar, A. W. Charney, A. Firpo-Betancourt, D. R. Mendu, J. Jhang, D. Reich, K. Sigel, C. Cordon-Cardo, M. Feldmann, S. Parekh, M. Merad, S. Gnjatic, An inflammatory cytokine signature predicts COVID-19 severity and survival. Nat. Med. 26, 1636–1643 (2020). 32839624 10.1038/s41591-020-1051-9 PMC7869028

[23] V. A. Koeken, L. C. J. de Bree, V. P. Mourits, S. J. Moorlag, J. Walk, B. Cirovic, R. J. Arts, M. Jaeger, H. Dijkstra, H. Lemmers, L. A. Joosten, C. S. Benn, R. van Crevel, M. G. Netea, BCG vaccination in humans inhibits systemic inflammation in a sex-dependent manner. J. Clin. Invest. 130, 5591–5602 (2020). 32692728 10.1172/JCI133935 PMC7524503

[24] G. Zizzo, P. L. Cohen, Imperfect storm: Is interleukin-33 the Achilles heel of COVID-19? Lancet Rheumatol. 2, e779–e790 (2020). 33073244 10.1016/S2665-9913(20)30340-4 PMC7546716

[25] L. Lu, H. Zhang, D. J. Dauphars, Y.-W. He, A potential role of interleukin 10 in COVID-19 pathogenesis. Trends Immunol. 42, 3–5 (2020). 33214057 10.1016/j.it.2020.10.012 PMC7605819

[26] S. K. P. Lau, C. C. Y. Lau, K. H. Chan, C. P. Y. Li, H. Chen, D. Y. Jin, J. F. W. Chan, P. C. Y. Woo, K. Y. Yuen, Delayed induction of proinflammatory cytokines and suppression of innate antiviral response by the novel Middle East respiratory syndrome coronavirus: Implications for pathogenesis and treatment. J. Gen. Virol. 94, 2679–2690 (2013). 24077366 10.1099/vir.0.055533-0

[27] W. G. Glass, H. F. Rosenberg, P. M. Murphy, Chemokine regulation of inflammation during acute viral infection. Curr. Opin. Allergy Clin. Immunol. 3, 467–473 (2003). 14612671 10.1097/00130832-200312000-00008

[28] Y. Yang, C. Shen, J. Li, J. Yuan, J. Wei, F. Huang, F. Wang, G. Li, Y. Li, L. Xing, L. Peng, M. Yang, M. Cao, H. Zheng, W. Wu, R. Zou, D. Li, Z. Xu, H. Wang, M. Zhang, Z. Zhang, G. F. Gao, C. Jiang, L. Liu, Y. Liu, Plasma IP-10 and MCP-3 levels are highly associated with disease severity and predict the progression of COVID-19. J. Allergy Clin. Immunol. 146, 119–127.e4 (2020). 32360286 10.1016/j.jaci.2020.04.027 PMC7189843

[29] Z. Zeng, H. Yu, H. Chen, W. Qi, L. Chen, G. Chen, W. Yan, T. Chen, Q. Ning, M. Han, D. Wu, Longitudinal changes of inflammatory parameters and their correlation with disease severity and outcomes in patients with COVID-19 from Wuhan, China. Crit. Care 24, 525 (2020). 32854750 10.1186/s13054-020-03255-0 PMC7450961

[30] E. Korpos, C. Wu, L. Sorokin, Multiple roles of the extracellular matrix in inflammation. Curr. Pharm. Des. 15, 1349–1357 (2009). 19355973 10.2174/138161209787846685

[31] S. Shi, M. Su, G. Shen, Y. Hu, F. Yi, Z. Zeng, P. Zhu, G. Yang, H. Zhou, Q. Li, X. Xie, Matrix metalloproteinase 3 as a valuable marker for patients with COVID-19. J. Med. Virol. 93, 528–532 (2020). 32603484 10.1002/jmv.26235 PMC7362036

[32] T. Ueland, J. C. Holter, A. R. Holten, K. E. Muller, A. Lind, G. K. Bekken, S. Dudman, P. Aukrust, A. M. Dyrhol-Riise, L. Heggelund, Distinct and early increase in circulating MMP-9 in COVID-19 patients with respiratory failure. J. Infect. 81, e41–e43 (2020). 32603675 10.1016/j.jinf.2020.06.061 PMC7320854

[33] K. R. Alkharsah, VEGF upregulation in viral infections and its possible therapeutic implications. Int. J. Mol. Sci. 19, 1642 (2018). 10.3390/ijms19061642 PMC6032371 29865171

[34] J. F. Korobelnik, A. Loewenstein, B. Eldem, A. M. Joussen, A. Koh, G. N. Lambrou, P. Lanzetta, X. Li, M. Lovestam-Adrian, R. Navarro, A. A. Okada, I. Pearce, F. J. Rodriguez, D. T. Wong, L. Wu, Guidance for anti-VEGF intravitreal injections during the COVID-19 pandemic. Graefes Arch. Clin. Exp. Ophthalmol. 258, 1149–1156 (2020). 32328757 10.1007/s00417-020-04703-x PMC7179379

[35] Y. Kong, J. Han, X. Wu, H. Zeng, J. Liu, H. Zhang, VEGF-D: A novel biomarker for detection of COVID-19 progression. Crit. Care 24, 373 (2020). 32576222 10.1186/s13054-020-03079-y PMC7309201

